# Supplementary material for: Active site specificity profiling datasets of matrix metalloproteinases (MMPs) 1, 2, 3, 7, 8, 9, 12, 13 and 14
Source: Data Brief. 2016 Feb 22;7:299–310. doi: 10.1016/j.dib.2016.02.036 (PMC4777984; doi:10.1016/j.dib.2016.02.036)
Supplement: Supplementary file 10 — Supplementary material [file mmc10.zip › WebPICS_hMMP1_G_1%/P3prime.html]

 

PICS results


|  |  |
| --- | --- |
| **P3prime\_A**  20 in 126 sites   15.9 %    effects > 10 perc. pnts.   (vice-versa in brackets)  P1\_C: 11.0 (44.1) |  |
| **P3prime\_K**  10 in 126 sites   7.9 %    effects > 10 perc. pnts.   (vice-versa in brackets)  P3\_V: 57.3 (35.9)   P2\_C: 16.8 (42.1)   P2\_L: 27.3 (17.1)   P1\_A: 11.3 (10.3)   P1\_H: 34.4 (49.2)   P2prime\_L: 17.3 (10.9) |  |
| **P3prime\_M**  4 in 126 sites   3.2 %    effects > 10 perc. pnts.   (vice-versa in brackets)  P2\_F: 38.9 (11.1)   P1\_H: 19.4 (11.1)   P1\_Y: 44.4 (25.4) |  |
| **P3prime\_N**  11 in 126 sites   8.7 %    effects > 10 perc. pnts.   (vice-versa in brackets)  P2\_F: 25.3 (19.9)   P2\_L: 14.6 (10.1)   P1\_R: 11.1 (13.5)   P2prime\_I: 26.9 (24.6) |  |
| **P3prime\_Q**  11 in 126 sites   8.7 %    effects > 10 perc. pnts.   (vice-versa in brackets)  P2\_L: 14.6 (10.1)   P2\_Q: 25.3 (19.9)   P1\_R: 11.1 (13.5)   P1\_Y: 21.7 (34.2)   P2prime\_L: 23.7 (16.3) |  |
| **P3prime\_T**  11 in 126 sites   8.7 %    effects > 10 perc. pnts.   (vice-versa in brackets)  P2\_A: 17.8 (16.3)   P1prime\_C: 11.9 (16.3)   P1prime\_M: 22.5 (41.3)   P2prime\_T: 19.4 (21.3) |  |
| **P3prime\_V**  16 in 126 sites   12.7 %    effects > 10 perc. pnts.   (vice-versa in brackets)  P2\_Y: 15.5 (20.6)   P2prime\_F: 14.0 (37.3)   P2prime\_T: 10.9 (17.3)   P2prime\_Y: 15.6 (62.3) |  |
